# Uretero-Jejunal Fistula: A Rare Cause of Acute Pyelonephritis

**DOI:** 10.7759/cureus.40824

**Published:** 2023-06-22

**Authors:** Adil S Mir, Varun Kesar, Vivek Kesar, Paul Yeaton

**Affiliations:** 1 Gastroenterology, Virginia Tech Carilion School of Medicine, Roanoke, USA; 2 Gastroenterology, Carilion Clinic, Roanoke, USA; 3 Internal Medicine, Virginia Tech Carilion School of Medicine, Roanoke, USA; 4 Gastroenterology and Hepatology, Carilion Clinic, Roanoke, USA

**Keywords:** upper urinary tract obstruction, obstructive hydronephrosis, perforated peptic ulcers, peptic perforation, enteric fistula, percutaneous endoscopic gastrostomy (peg), jejunal perforation, axios stenting, acute pyelonephritis, complicated peptic ulcer disease

## Abstract

Penetrating peptic ulcers often lead to severe complications. The development of uretero-enteric fistulas is rare and can be challenging to diagnose and treat. Here, we present the case of a 41-year-old patient who previously underwent gastrojejunostomy for superior mesenteric artery syndrome and developed a peptic jejunal ulcer, leading to a uretero-jejunal fistula and finally causing acute pyelonephritis. The patient was managed with a multidisciplinary approach including medical therapy and endoscopic and radiologic interventions.

## Introduction

Uretero-enteric fistulas represent a rare complication of peptic ulcer disease and are often associated with recurrent and/or chronic urinary tract infections [1]. They account for nearly 1% of urinary tract fistulas [2]. Most common causes include trauma, ischemia, chronic inflammatory bowel disease, complicated diverticulitis, malignancies, radiation therapy, and chronic renal transplant rejection [2,3,4]. Depending on the location, different segments of the small bowel can be affected [5]. Such fistulas can be challenging to manage and often require a multidisciplinary approach for comprehensive management. Here, we report a unique case in which a patient developed a rare complication of the uretero-jejunal fistula with a previous history of gastrojejunostomy for superior mesenteric artery (SMA) syndrome. This fistula was likely multifactorial in etiology and was managed using a multidisciplinary team approach.

## Case presentation

A 41-year-old female with a history of cervical cancer requiring chemotherapy and radiation therapy, complicated by a rectovaginal fistula requiring end colostomy with a Hartmann pouch more than 10 years ago, presented with nausea and vomiting, poor oral intake, and weight loss of 40 pounds over the past few months. Computed tomography (CT) of the abdomen and pelvis with intravenous (IV) contrast showed no findings suggestive of recurrent cancer. However, it showed dilation of the stomach and proximal duodenum to the level of the transverse duodenum where it crosses between the aorta and SMA, and the patient was diagnosed with SMA syndrome. The patient refused a naso-jejunal tube placement. Other options were discussed with the patient, and after detailed informed discussions, she eventually underwent endoscopic ultrasound (EUS)-guided gastrojejunostomy with lumen-apposing metal stent (LAMS) AXIOS^TM^ placement (Boston Scientific, Natick, MA).

**Figure 1:**
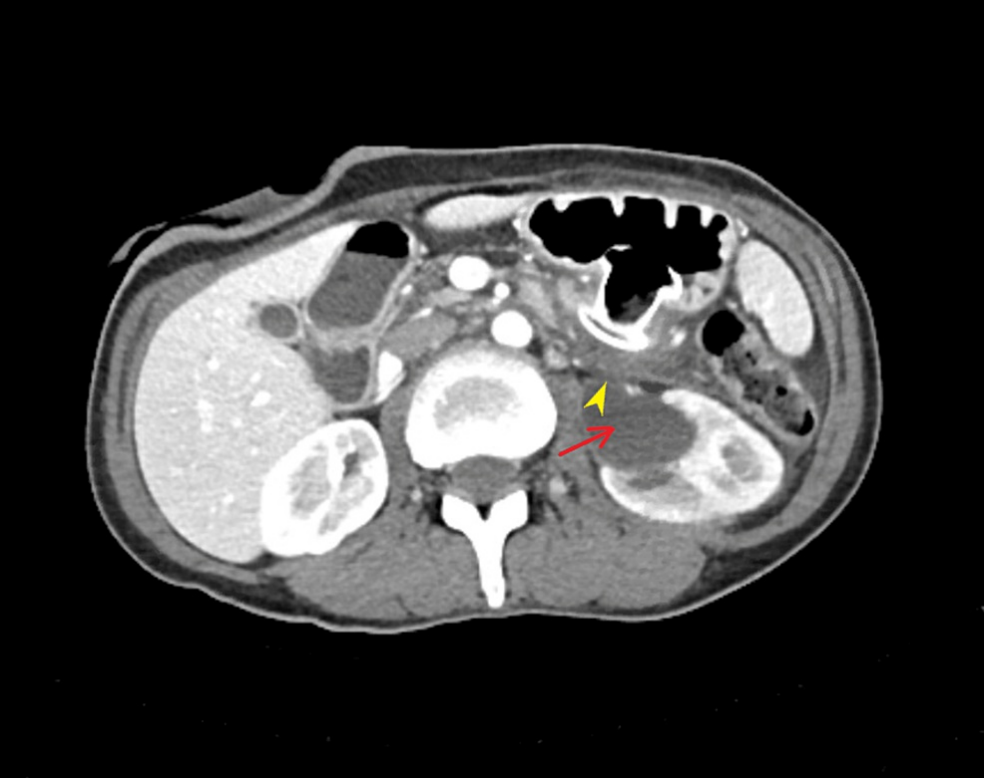
CT scan of the abdomen with IV contrast showing left-sided hydronephrosis (red arrow) and close apposition of the left ureter with jejunum (yellow arrowhead)

Subsequently, after a month, the patient presented with left-sided flank pain associated with fever and chills. She was noted to have new leukocytosis of 18.9 K/uL (reference range: 4-10.5 K/uL). Urinalysis was suggestive of urinary tract infection (>100 WBCs/high-power field). Urine culture showed >100,000 COL/mL *Escherichia coli* sensitive to ceftriaxone. CT of the abdomen and pelvis with IV contrast revealed a patchy perfusion of the parenchyma of the left kidney, interval development of left-sided hydronephrosis (Figure 1, red arrow), and obstruction of the proximal left ureter due to abnormally enlarged left periaortic lymph nodes. Then, the AXIOS^TM^ stent interposed between the greater curvature of the stomach and small bowel distal to the ligament of Treitz. The patient was started on treatment with IV ceftriaxone.

**Figure 2:**
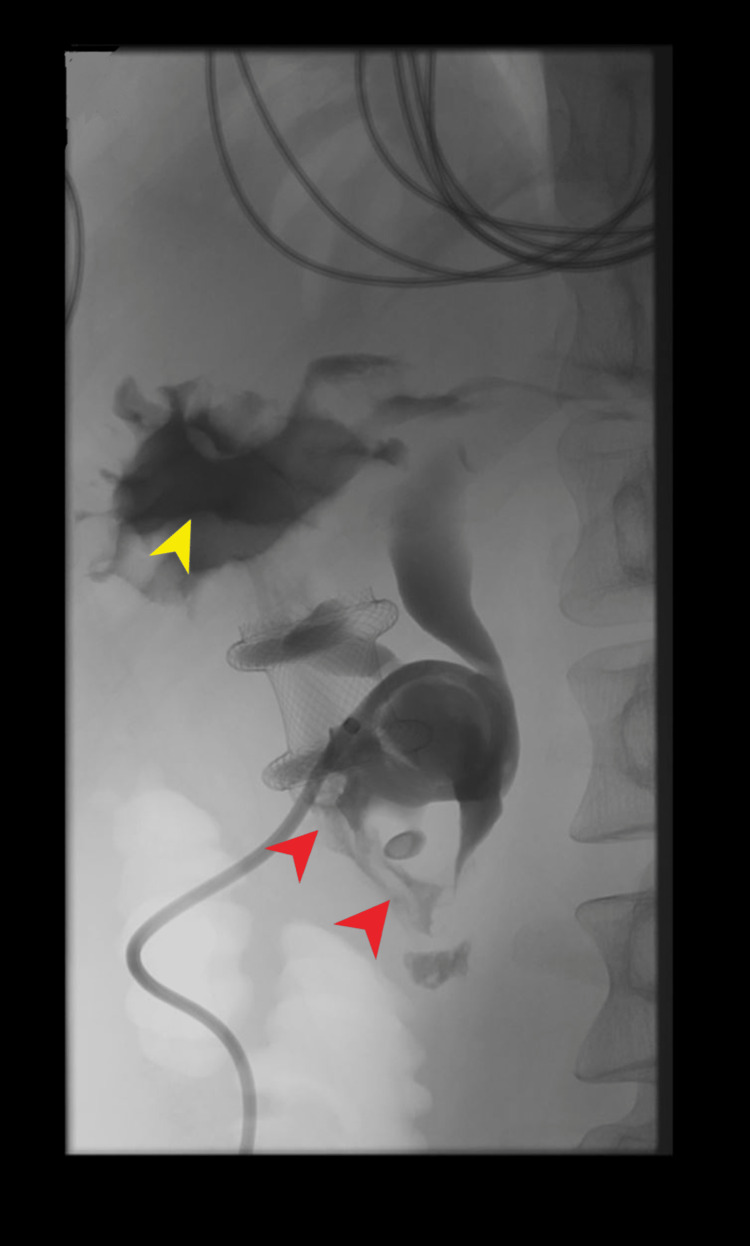
Nephrostogram showing the flow of contrast from the proximal left ureter to the AXIOS stent (red arrowheads) and stomach (yellow arrowhead)

The patient was admitted for the management of left-sided pyelonephritis, started on IV ceftriaxone, and underwent left-sided nephrostomy tube placement by interventional radiology (IR). However, due to a concern on leaks from the proximal left ureter, the patient underwent nephro-ureteral catheter placement by IR. Further review of the CT scan also revealed close apposition of the left ureter with jejunum (Figure 1, yellow arrowhead). During the procedure, a nephrostogram was obtained, which showed a flow of contrast from the proximal left ureter to the AXIOS^TM^ stent and stomach (Figure 2).

**Figure 3:**
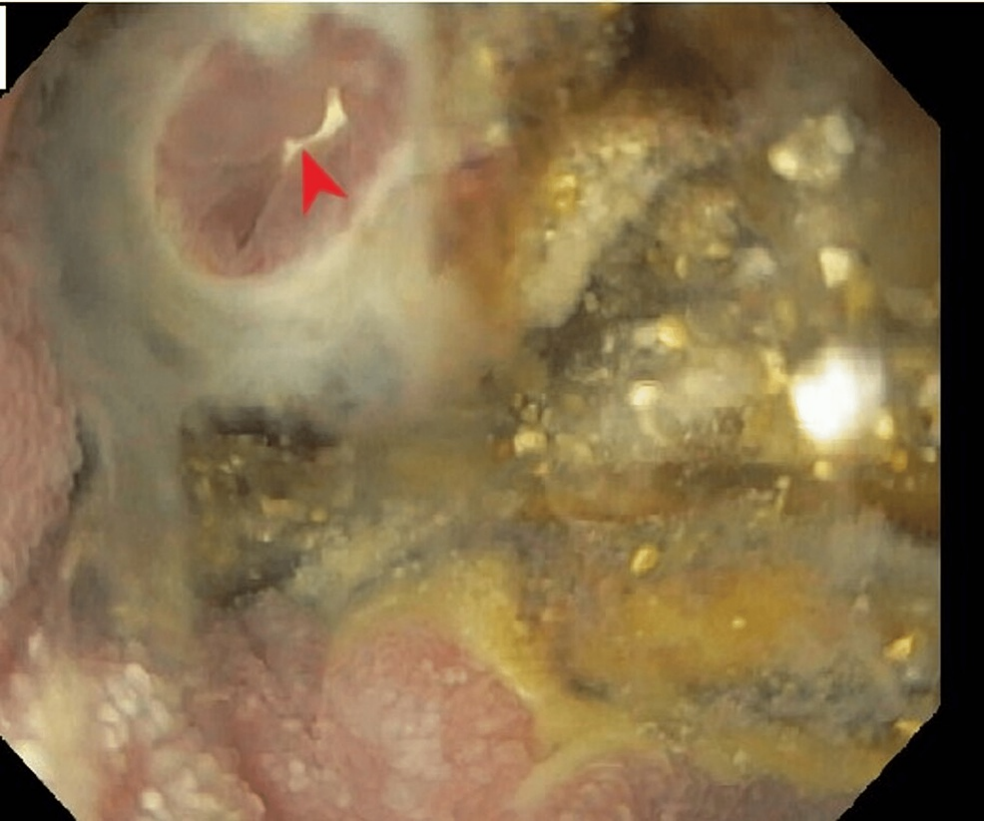
EGD showing a jejunal ulcer with an opening at the base (red arrowhead) EGD: esophagogastroduodenoscopy

**Figure 4:**
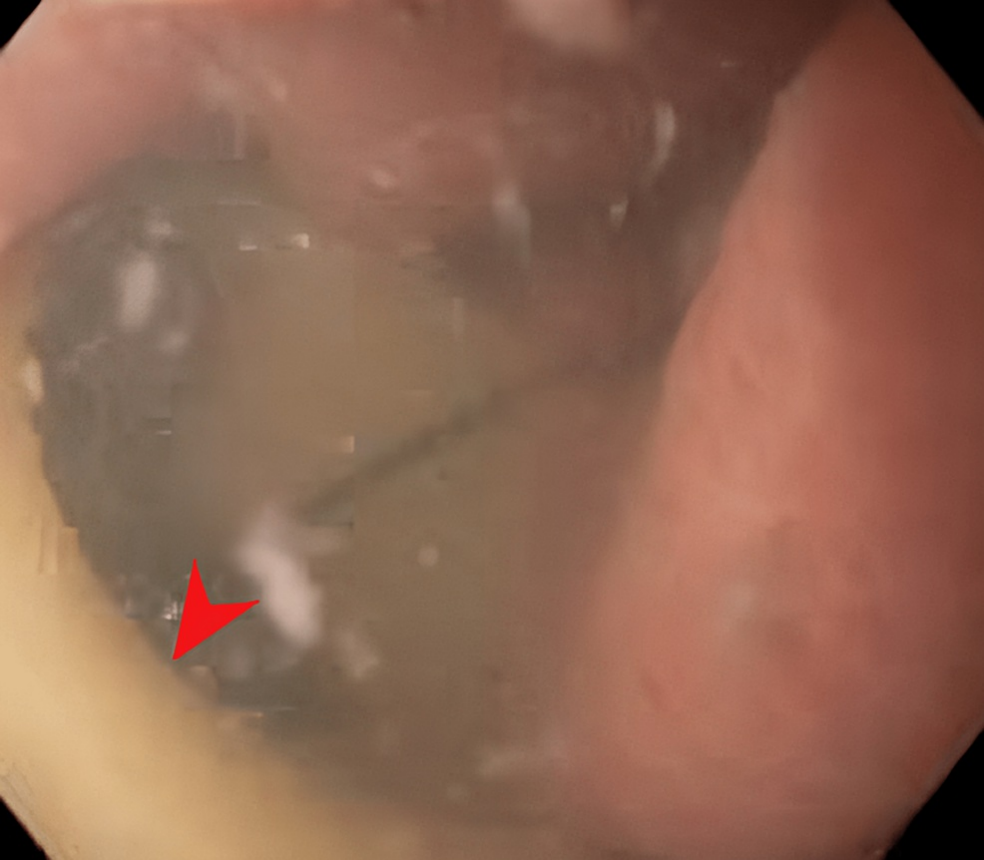
Endoscopic image during EGD showing a mucosal opening, through which a white ureteral stent was seen through the ulcer base (red arrowhead) EGD: esophagogastroduodenoscopy

Hospital course was further complicated by a melenic output (from the colostomy) and acute blood loss anemia (hemoglobin count downtrended to 6.8 g/dL from the previous baseline of around 10-11 g/dL; reference range: 12-16 g/dL) requiring packed red blood cell transfusion. The patient underwent an esophagogastroduodenoscopy (EGD), which showed multiple superficial erosions and a jejunal ulcer with an opening at the base (Figure 3). During the endoscopic procedure, the mucosal opening was assessed using a ball-tip catheter, through which a white ureteral stent was seen through the ulcer base (Figure 4).

During the endoscopic procedure, the LAMS stent was removed, and a percutaneous endoscopic gastrojejunostomy (PEG-J) tube was placed for nutrition bypassing the ulcer site. The patient was continued with high-dose acid suppression, subsequently started on tube feeding the next day, and finally discharged with a 14-day course of oral antibiotics, which she tolerated well.

On outpatient follow-up, the patient was noted to have clinical improvement and weight gain. Follow-up EGD after six months showed endoscopic evidence of ulcer healing. Surveillance positron emission tomography (PET) scan showed no evidence of metabolically active disease. However, about a year later, she presented with worsening cachexia, abdominal distension, fever, and encephalopathy, and CT scan of the abdomen showed free intraperitoneal air. Due to concern of viscus perforation, the surgical team was consulted, but it was deemed that the patient was at high risk for open surgical intervention. The palliative team was subsequently consulted, and the patient's next of kin and family elected for no aggressive interventions and comfort care only. The patient was given comfort care and eventually passed away after three days.

## Discussion

The possible mechanism for erosions and ulcer formation is most likely due to the delivery of an acidic chyme load directly into the jejunum (via gastrojejunostomy) without neutralization from the bicarbonate-rich pancreatic secretions (because the acidic chyme bypasses the duodenum without stimulating the pancreas) [6]. Peptic ulcers can penetrate nearby organs leading to multiple complications depending on the site, which sometimes can even be fatal. Bleeding, perforation, penetration, and gastric outlet obstruction are the most common complications of peptic ulcers [2,3,4,7].

Our patient represents a unique case of ulcer penetration into the proximal left ureter. We recommend aggressive acid suppression for ulcer prevention in such cases. Unfortunately, non-compliance to proton pump inhibitors led to unopposed acid secretion in the above-mentioned patient leading to ulcer formation. Alternatively, some degree of ischemia due to pressure on the bowel wall from close apposition due to hydronephrosis can also be suggested. Our patient also had a history of chemoradiation therapy for cervical cancer, which could, at least in part, also contribute to the fistula formation.

Surgical management options include reconstructive procedures, uretero-ureterostomy, small bowel interposition, native ureter-pyelostomy, or ureter-ileostomy loop. However, open surgical procedures have the risk of high morbidity, and interventional radiology-guided minimally invasive procedures, such as nephro-ureteral stent placement, have shown good promise in the management of uretero-enteric fistulas [2]. Antibiotics should be used in cases of active infections. However, in severe, non-healing, or recurrent cases, surgical correction of the fistula may be needed.

## Conclusions

The possibility of this rare yet potentially life-threatening complication should always be considered by clinicians while taking care of patients with complicated health conditions. As of now, the literature on the exact incidence of this rare complication remains limited. However, with the advent of readily available endoscopic and radiologic modalities, uretero-enteric fistulas can be definitively diagnosed and managed. Future studies examining the incidence, diagnosis, and treatment modalities of this rare complication will help educate clinicians to maintain a high level of care in diagnosing it and hence provide a timely treatment.
